# Interactions of retinoids with the ABC transporters P-glycoprotein and Breast Cancer Resistance Protein

**DOI:** 10.1038/srep41376

**Published:** 2017-02-01

**Authors:** Szabolcs Tarapcsák, Gábor Szalóki, Ágnes Telbisz, Zsuzsanna Gyöngy, Krisztina Matúz, Éva Csősz, Péter Nagy, Imre J. Holb, Ralph Rühl, László Nagy, Gábor Szabó, Katalin Goda

**Affiliations:** 1 Department of Biophysics and Cell Biology, University of Debrecen, Debrecen, H-4002 Egyetem tér 1, P.O.B. 400, Hungary; 2 Institute of Enzymology, Research Centre for Natural Sciences, Hungarian Academy of Sciences, Budapest, H-1117 Magyar tudósok körútja 2, P.O.B. 286, Hungary; 3 Department of Biochemistry and Molecular Biology, University of Debrecen, Debrecen, H-4002 Egyetem tér 1, P.O.B. 400, Hungary; 4 Institute of Horticulture, University of Debrecen, Debrecen, H-4015 Böszörményi út 138, P.O.B. 400, Hungary; 5 Plant Protection Institute, Centre for Agricultural Research, Hungarian Academy of Sciences, Budapest, H-1525 Hermann Ottó út 15, P.O.B. 525, Hungary; 6 MTA-DE, Public Health Research Group of the Hungarian Academy of Sciences, Faculty of Public Health, University of Debrecen, Debrecen, H-4028 Kassai út 26, P.O.B. 400, Hungary

## Abstract

Retinoids – derivatives of vitamin A – are important cell permeant signaling molecules that regulate gene expression through activation of nuclear receptors. P-glycoprotein (Pgp) and ABCG2 are plasma membrane efflux transporters affecting the tissue distribution of numerous structurally unrelated lipophilic compounds. In the present work we aimed to study the interaction of the above ABC transporters with retinoid derivatives. We have found that 13-*cis*-retinoic acid, retinol and retinyl-acetate inhibited the Pgp and ABCG2 mediated substrate transport as well as the substrate stimulated ATPase activity of these transporters. Interestingly, 9-*cis*-retinoic acid and ATRA (all-*trans* retinoic acid), both are stereoisomers of 13-*cis*-retinoic acid, did not have any effect on the transporters’ activity. Our fluorescence anisotropy measurements revealed that 13-*cis*-retinoic acid, retinol and retinyl-acetate selectively increase the viscosity and packing density of the membrane. Thus, the mixed-type inhibition of both transporters by retinol and ABCG2 by 13-*cis-*retinoic acid may be the collective result of direct interactions of these retinoids with the substrate binding site(s) and of indirect interactions mediated by their membrane rigidifying effects.

P-glycoprotein (Pgp, MDR1, ABCB1) and Breast Cancer Resistance Protein (BCRP, MXR, ABCG2) are members of the ATP-binding-cassette (ABC) transporter family characterized by their evolutionary conserved ATP-binding domains. ABC transporters form one of the largest protein families and are present in every organism from bacteria to human (for reviews see refs 1, 2, 3). In higher eukaryotes several ABC transporters have important functions in drug metabolizing and drug excreting organs, e.g. in the liver and the kidney, and also play a role in the formation of tissue barriers, including the intestinal epithelium, blood-brain, blood-placenta and blood-testis barriers, suggesting that these proteins are important determinants of the pharmacokinetics of various chemotherapeutic compounds^4^. Since Pgp and ABCG2 is expressed in the apical membrane of enterocytes, they affect the intestinal absorption of nutrients, drugs, food additives and xenobiotics^5^. In addition, overexpression of Pgp and ABCG2 has also been demonstrated in chemotherapy resistant tumors of various tissue origins (for reviews see refs 6, 7, 8, 9). Of note, ABCG2 is also involved in rather specific physiological functions e.g. transport of urate^10^, androgens and estrogens^11^, while such roles have not yet been identified for Pgp.

A common structural feature of active ABC transporters is that they contain two nucleotide binding domains (NBDs) responsible for binding and hydrolysis of ATP and at least two transmembrane domains (TMDs) forming the drug binding pocket and the drug translocation pathway. Pgp and ABCG2 have particularly wide and partially overlapping substrate spectra without any significant sequence similarity in the TMDs of the two transporters^12^. Mutagenesis studies as well as photoaffinity labeling in conjunction with mass spectrometry suggest that the drug-binding pocket of Pgp consists of multiple drug interaction sites located at the interface between the two TMDs involving both halves of the protein^13^,^14^,^15^,^16^. Analogously, it seems reasonable that the drug-binding pocket of ABCG2 is collectively formed by the TMDs of the dimerizing partners. Based on high-resolution crystal structures of Pgp as well as biophysical and biochemical studies, it is suggested that the substrate binding cavity is accessible from both the cytoplasm and the inner membrane leaflet^17^. Similarly to Pgp, ABCG2 also seems to export its substrates, like mitoxantrone, directly from the plasma membrane^18^. Therefore, the extent of partitioning of substrates into the membrane is important for their interaction with Pgp and ABCG2, because it essentially controls their effective concentration presented to the transporter (for a review see ref. 19).

Retinoids play an essential role in a number of physiological mechanisms including cell survival and transcriptional regulation of cell differentiation and proliferation. Retinol is present in the plasma at 1–2 micromolar concentration^20^, while its natural metabolites including all-*trans* retinoic acid (ATRA), 9-*cis*-retinoic acid or 13-*cis*-retinoic acid (isotretionin) are present in the plasma at nanomolar concentrations and can regulate the expression of specific genes upon activation of nuclear receptors including RAR-RXR heterodimers, PPARs and VDR^21^,^22^,^23^. Certain retinoid derivatives including ATRA and 13-*cis*-retinoid acid are used in chemotherapy for the treatment of neuroblastoma or squamous cell carcinoma at much higher concentrations resulting in 10–20 micromolar plasma concentrations^24^,^25^,^26^. Retinyl-acetate and retinyl-palmitate are widely applied as food additives, and are also used as a component of anti-ageing cosmetics^27^. Retinyl-acetate also seems promising in the treatment of certain degenerative diseases of the retina^28^. According to literature data certain retinoid derivatives show uneven distribution between the placenta and the embryo, raising the possibility that active transporters might be involved in their transport^29^. Since Pgp and ABCG2 are expressed in the placenta, they might have roles in the protection of the fetus from xenobiotics and teratogens such as retinoids^30^.

In this study we aimed to elucidate the interactions of different retinoid compounds of physiological or medical relevance with human multidrug transporter proteins Pgp and ABCG2, to answer the question if they might be ligands of these proteins.

## Results

### Certain retinoids inhibit the drug transport activity of Pgp and ABCG2 in live cells

**Figure 1:**
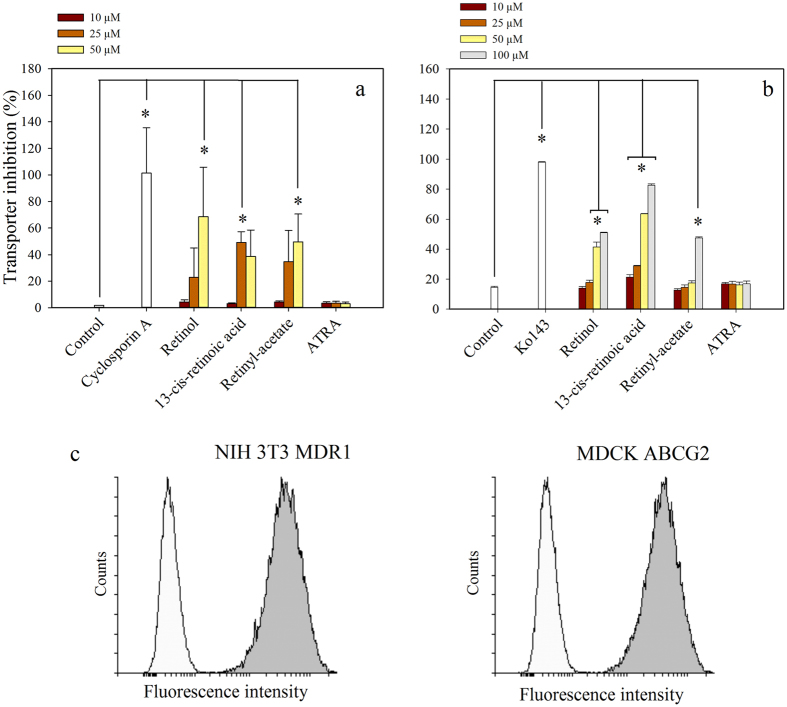
Effect of retinoid derivatives on the transport activity of Pgp (**a**) and ABCG2 (**b**) expressed in NIH 3T3 and MDCK cells, respectively. Cells were pre-treated with retinoids or transporter inhibitors (cyclosporin A or Ko143) for 20 min at 37 °C and then the NIH 3T3/NIH 3T3 MDR1 cell line pair was stained with 500 nM calcein-AM for 20 min (**a**), while the MDCK/MDCK ABCG2 cell pair (**b**) was stained with 5 μM mitoxantrone for 40 min at 37 °C. The calcein or mitoxantrone accumulation of the transporter expressing cells is shown as a percentage of that of the transporter negative cells. Mean ± SD values of three independent experiments are shown. The expression levels of the transporters were determined by direct immunofluorescence using Alexa 647 conjugated 15D3 mAb for Pgp and 5D3 mAb for ABCG2 (**c**). (*significant difference compared to the control, P < 0.05 by ANOVA, Holm-Sidac post-hoc analysis).

We have studied the effects of retinoids on the transport activity of Pgp and ABCG2 using cell lines expressing these pumps at high level, as it is demonstrated by direct immunofluorescence (Fig. 1c). Calcein-AM was applied to study the transport function of Pgp, while mitoxantrone was used to visualize ABCG2-mediated transport. Our data show that retinol, 13-*cis*-retinoic acid and retinyl-acetate increased the cellular accumulation of the fluorescent transporter substrates in a concentration dependent manner in Pgp and ABCG2 expressing cells alike (Fig. 1a,b). When applied at relatively high concentrations (25–100 μM, see Fig. 1a,b), the effect of these retinoid derivatives was comparable to that of the specific transporter inhibitors, however they did not have any effect on the transporter negative cells (Supplementary Fig. S1).

### Effect of retinoids on the ATPase activity of Pgp and ABCG2

**Figure 2:**
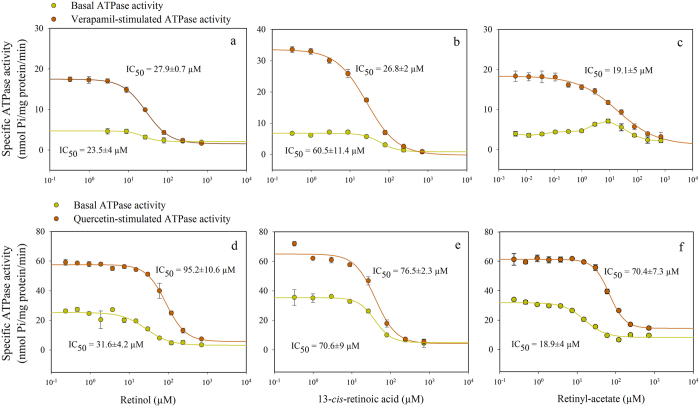
Dose-response curves demonstrating the effects of retinoids on the ATPase activity of Pgp and ABCG2. Pgp (**a**,**b**,**c**) and ABCG2 (**d**,**e**,**f**) expressing Sf9 cell membrane preparations were pre-incubated with retinoids for 10 min at 37 °C and then further incubated in the presence of 3 mM ATP/Mg^2+^ for 25 min. The substrate-dependent ATPase activity of Pgp was measured in the presence of 40 μM verapamil, while the activity of ABCG2 was induced by 10 μM quercetin. Representative graphs are shown out of three independent experiments performed in triplicates (data points are their means ± SD). Data points were fitted with a four parameter sigmoidal dose-response relationship (R^2^ ≥ 0.98, where R^2^ is the coefficient of determination; see Materials and Methods) to determine the IC_50_ values. IC_50_ values are given as means ± SEM from 3 independent measurements.

Since retinoids might be isomerized or converted to other retinoid derivatives in live cells^31^, in further experiments we applied *Spodoptera frugiperda (Sf9*) membrane preparations expressing the examined transporters (see Supplementary Fig. S2). We measured the effects of the retinoids on the basal and the substrate-stimulated ATPase activity of the transporters^32^. Due to low expression of endogenous ATPases and high expression of human ABC transporters, the Sf9 cell membrane preparations exhibited high specific ATPase activities^33^. The substrate concentrations required for the maximum stimulation were determined: treatment with 40 μM verapamil brought about a 4-fold stimulation of the ATPase activity of Pgp (approximately 20 nmol Pi/mg protein/min), while 10 μM quercetin induced about a 2-fold stimulation of ABCG2 activity up to 60–70 nmol Pi/ mg protein/min (Fig. 2).

Retinyl-acetate slightly stimulated the basal ATPase activity of Pgp (Fig. 2c, yellow symbols). However, we could not demonstrate its Pgp substrate character in cytotoxicity measurements since Pgp^+^ and Pgp^−^ cells exhibited identical cytotoxicity profile (see Supplementary Fig. S3).

In accordance with the substrate accumulation assays carried out in live cells (Fig. 1), retinyl-acetate hampered the substrate-stimulated ATPase activity of Pgp and ABCG2 (Fig. 2c,f, brown symbols), while retinol and 13-*cis*-retinoic acid inhibited both the basal- and the substrate-stimulated ATPase activity of either transporters (Fig. 2a,b,d,e). From the dose-response curves, we calculated the IC_50_ values for the basal and the substrate stimulated ATPase activities as it shown in Fig. 2.

Several other physiological retinoid derivatives including ATRA, 9-*cis*-retinoic acid, retinyl-propionate and retinyl-palmitate were tested in our drug accumulation and ATPase activity measurements, but none of them had any effect on the transport or ATPase activity of Pgp and ABCG2 (see Fig. 1 and Supplementary Table S1).

### Cellular accumulation of retinoids

**Figure 3:**
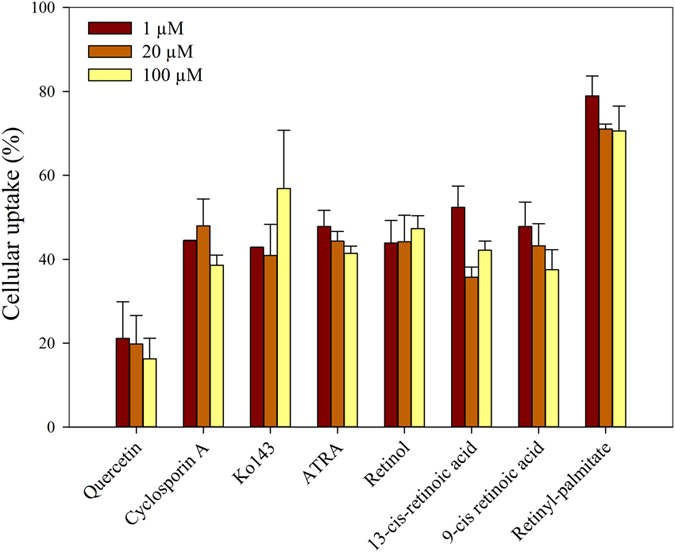
Cellular uptake of retinoids and transporter modulators. Samples containing retinoids or transporter inhibitors were incubated with NIH 3T3 cells (1 × 10^6^/ml) for 30 min at 37 °C, then the cells were removed by 5 min centrifugation at 400 × g. Cellular uptake of the examined compounds was determined by calculating the ratio of the absorbances measured in the supernatants before and after incubation with cells. (n = 3, mean ± SD).

13-*cis*-retinoic acid and retinol inhibited both Pgp and ABCG2. Intriguingly however, ATRA (*all-trans* retinoic acid) and 9-*cis*-retinoic acid differing only in the position of the *cis* double bond from 13-*cis*-retinoic acid did not affect the transporters’ activity. We supposed that low membrane partitioning and weak cellular accumulation might be responsible for the lack of effect. Although the octanol-water partition coefficient of each retinoid was found to be very high as it is shown in Supplementary Table S2, membrane partitioning might also be affected by the shape of the molecules. Since the cellular accumulation of drugs is dependent on their membrane partitioning, we estimated the fraction of retinoids accumulated by Pgp and ABCG2 negative NIH 3T3 cells (Fig. 3). We have found that the cellular accumulation of the examined Pgp and ABCG2 substrates/modulators and retinoids correlated with their octanol-water partition coefficient in a large concentration range (compare the data in Fig. 3 and Supplementary Table S2).

### Effects of retinoids on the fluorescence anisotropy of DPH and TMA-DPH

In view of the strong lipophilicity of retinoids (see Supplementary Table S2) as well as the intimate association of Pgp and ABCG2 with the lipid bilayer in which they are embedded and from which they harvest their substrates, we examined the effects of retinoids on the fluidity and packing order of the membrane. We carried out fluorescence anisotropy measurements using fluorescent membrane probes, DPH and TMA-DPH. DPH is an apolar molecule that partitions into the acyl-chain region of the membrane, while TMA-DPH is a cationic derivative of DPH^34^. Since its positive charge is anchored at the membrane interface the average fluorophore of TMA-DPH is localized closer to the membrane surface by about 3–4 Å compared to DPH. Thus, the measured TMA-DPH and DPH fluorescence anisotropy values reflect the fluidity and packing order in slightly different depths of the membrane^35^,^36^.

**Figure 4:**
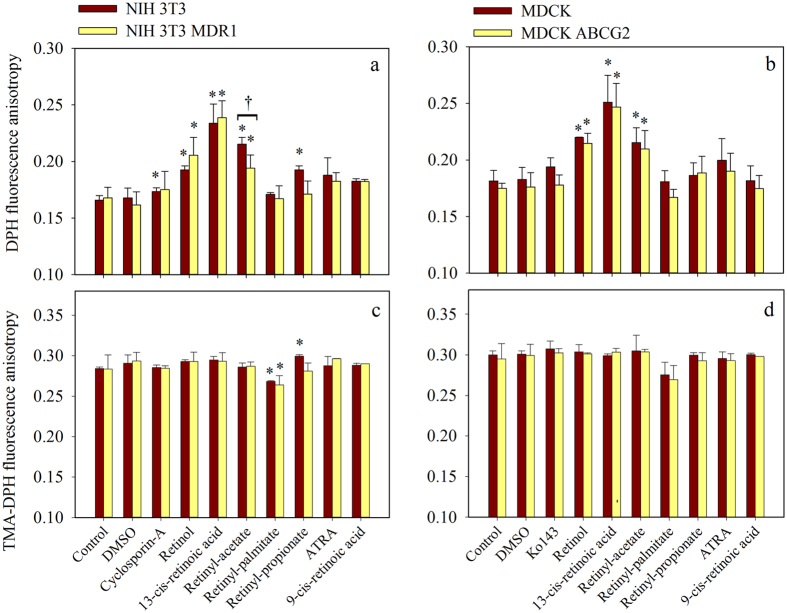
Effects of retinoids on the DPH (**a**,**b**) and TMA-DPH (**c**,**d**) fluorescence anisotropies in Pgp^+^ and Pgp^−^ NIH 3T3 cells (**a**,**c**), and ABCG2 expressing and non-expressing MDCK cells (**b**,**d**). Cells were pre-treated with retinoids applied at 100 μM concentration or transporter inhibitors (10 μM cyclosporin (for Pgp) or 2 μM Ko143 (for ABCG2)) for 10 min at 37 °C and then further incubated with 2 μM DPH or TMA-DPH for another 20 min at room temperature. Fluorescence anisotropy values were measured at 37 °C. (*significant difference compared to control, ^†^significant difference between drug-sensitive and -resistant cells (n = 3, mean ± SD, ANOVA, Holm-Sidac post-hoc analysis, P < 0.05)).

The steady-state fluorescence anisotropy of DPH was in the range of 0.14 and 0.18 (Fig. 4a,b), while the fluorescence anisotropy of TMA-DPH changed between 0.28 and 0.30 for the different cell lines used in this study (Fig. 4c,d). The measured anisotropy values were slightly cell line specific, but independent of the expression of the transporters, in accordance with previous data^37^. Interestingly, retinol, 13-*cis*-retinoic acid and retinyl-acetate, the retinoids that proved capable of Pgp and ABCG2 inhibition, increased the DPH fluorescence anisotropy values significantly in the case of all tested cell lines, while the other retinoids investigated had no significant effect (Fig. 4). Retinol and 13-*cis*-retinoic acid modified the DPH anisotropy similarly in the transporter expressing and non-expressing cell lines suggesting that the presence or activity of the transporters do not affect the distribution of these retinoids in the membrane. In contrast, retinyl-acetate had significantly lower effect on the DPH fluorescence anisotropy of the Pgp^+^ NIH 3T3 MDR1 cell line compared to its Pgp^−^ counterpart (Fig. 4a) apparently because of its altered sequestration in the membrane of Pgp expressing cells.

The examined retinoids did not affect the TMA-DPH fluorescence anisotropy values except for retinyl-palmitate that decreased the TMA-DPH anisotropies significantly in the NIH 3T3 cell pair.

In control experiments neither CsA a competitive Pgp-specific modulator, nor the ABCG2-specific inhibitor Ko143 had any effect on the anisotropy values of DPH and TMA-DPH, supporting that they do not affect the overall membrane fluidity.

### Effects of retinoids on the substrate stimulated ATPase activity of Pgp and ABCG2

**Figure 5:**
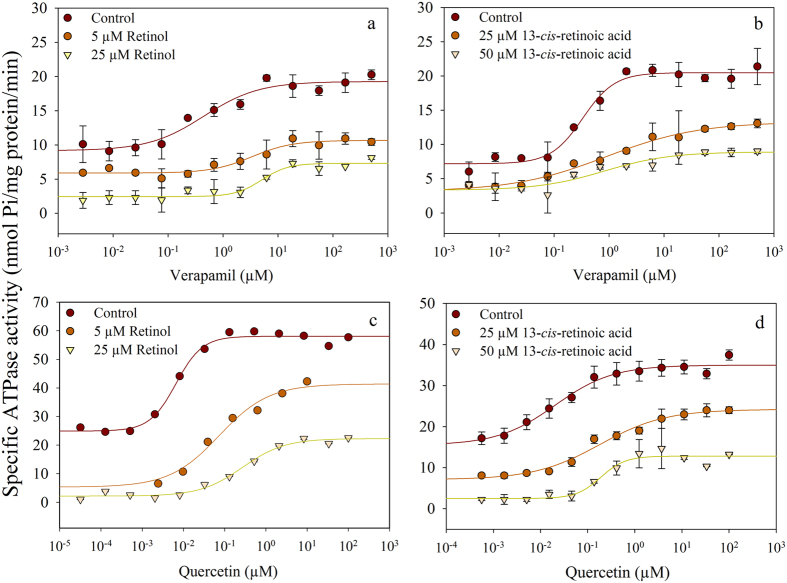
Inhibition of the substrate stimulated ATPase activity of Pgp and ABCG2 by retinol and 13-*cis*-retinoic acid. The ATPase activity of Pgp (**a**,**b**) and ABCG2 (**c**,**d**) was stimulated by 40 μM verapamil and 10 μM quercetin, respectively. Representative data are shown out of three independent experiments performed in triplicate (data points are means ± SD). The curves were obtained by fitting the data points with a modified form of the Michaelis-Menten equation (see Materials and Methods, (R^2^ ≥ 0.98)).

**Figure 6:**
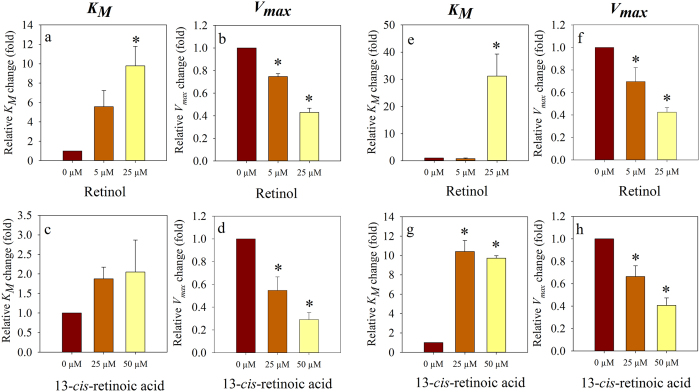
Changes of apparent *K*_*M*_ and *v*_*max*_ values of the Pgp- (**a**–**d**) and ABCG2-mediated (**e**–**h**) ATPase activity in response to retinol and 13-*cis*-retinoic acid treatment. The apparent *K*_*M*_ and *v*_*max*_ values were obtained by curve-fitting to a modified form of the Michaelis-Menten equation (see Materials and Methods). The ATPase activity of Pgp (**a**,**b**,**c** and **d**) and ABCG2 (**e**,**f**,**g** and **h**) was stimulated by 40 μM verapamil and 10 μM quercetin, respectively. (*Significant difference compared to control (n = 3, mean ± SEM, ANOVA, Holm-Sidac post-hoc analysis, P < 0.05)).

The inhibitory effect of retinoids observed in the previous experiments may be attributed to the allosteric inhibition of the transporter, probably related to structural changes of the membrane brought about by retinoids, or to a competitive mechanism due to direct interaction of retinoids with the substrate binding site(s). In order to resolve the above problem we analyzed how the kinetic parameters (*K*_*M*_ and *v*_*max*_) of the substrate stimulated ATPase activity are influenced by retinol and 13-*cis*-retinoic acid, the agents that inhibit both the basal and the substrate stimulated ATPase activities of both transporters. In these experiments the ATPase activity of Pgp was stimulated by verapamil, while the activity of ABCG2 was induced by quercetin similarly to the previous experiments shown in Fig. 2. Fitting the data points obtained at different verapamil/quercetin concentrations with a modified form of the Michaelis-Menten equation we have found that retinol increased the *K*_*M*_ and decreased the *v*_*max*_ values of both transporters (Figs 5a,c and 6a,b,e,f) supporting a mixed-type transporter inhibition. 13-*cis*-retinoic acid also showed mixed-type transporter inhibition of ABCG2 (Figs 5d and 6g,h), however in case of Pgp it did not induce statistically significant *K*_*M*_ increase while decreased the *v*_*max*_ (Figs 5b and 6c,d), suggesting that it inhibits Pgp only non-competitively.

## Discussion

We have shown that retinol, 13-*cis*-retinoic acid and retinyl-acetate inhibited both the Pgp- and ABCG2-mediated substrate transport (Fig. 1). Because of the difficulty of directly assessing the binding and transport of highly lipophilic drugs by membrane transporters^19^, the interaction of retinoids with Pgp and ABCG2 was further studied in ATPase activity measurements. In these experiments we have found that retinyl-acetate slightly stimulated the basal ATPase activity of Pgp (Fig. 2) raising the possibility that it is a transport substrate of Pgp. In accordance with it our DPH anisotropy measurements also indicated altered membrane distribution of retinyl-acetete in Pgp^+^ cells (Fig. 4a). However, we observed identical cytotoxicity profile of retinyl-acetate in Pgp^+^ and Pgp^−^ cells (see Supplementary Fig. S3), suggesting that even if it interacts with Pgp as a substrate, the transporter cannot cope with its passive influx.

Of note, 13-*cis*-retinoic acid inhibited both Pgp and ABCG2, while its stereoisomer ATRA (*all-trans* retinoic acid) and 9-*cis*-retinoic acid differing only in the position of the *cis* double bond did not affect the transporters’ activity (see Figs 1 and 2 and Supplementary Table S1). In view of the wide substrate spectrum of Pgp and ABCG2, this stereospecific interaction is striking and might provide further insight into the working mechanism of these transporters. In line with our observations, stereoisomers of a cyclic oligopeptide interact differently with Pgp based on crystallography data and homology modeling: QZ59-RRR binds at only one site located at the center of the Pgp molecule between TM6 and TM12, while its stereoisomer QZ59-SSS binds at two sites per Pgp^38^. Similarly, stereo-chemical differences have been observed in the case of the interaction of *cis*- and *trans-* stereoisomers of flupentixol with Pgp. Both stereoisomers of flupentixol inhibit Pgp-mediated drug transport and reverse drug resistance. They have equal binding affinity to Pgp, but they have opposite effects on the rate of ATP hydrolysis and photo affinity labeling of Pgp with the substrate analogue [125I]IAAP^39^. Taken together, previous studies established stereospecific differences between ligands in their mode of interaction with Pgp. In contrast, we observed that the recognition of certain retinoid derivatives by Pgp and ABCG2 is stereo-selective.

Stereo-selective recognition of the ligands might occur at the level of the drug binding sites or allosteric sites of the transporters or alternatively, at the level of the plasma membrane from where the substrates and modulators probably reach the drug binding site(s). The lipophilicity of the stereoisomers based on their octanol-water partition coefficients (LogP_ow,_ see Supplementary Table S2) is similarly high and they exhibit strong cellular accumulation (Fig. 3) that correlates with their LogP_ow_, thus different extent of partitioning into the membrane does not seem to explain their behavior. However, distinct intramembrane localization of the stereoisomers may explain their different behavior. In agreement with this idea, Widomska and Subczynski have demonstrated that *cis* and *trans* isomers of zeaxanthin have different orientations in dimyristoyl phosphatidylcholine bilayer membranes and thus can modify the biophysical membrane properties, including the hydrophobicity and membrane fluidity at different depth of the membrane^40^. In accordance with this report our fluorescence anisotropy measurements revealed that the retinoid derivatives interacting with the transporters retinyl-acetate, 13-*cis*-retinoic acid and retinol selectively increase the membrane viscosity and packing density in the depth of the membrane monitored by DPH, while ATRA and 9-*cis*-retinoic acid did not have any effect (Fig. 4). These results imply that the transmembrane orientation of the retinoic acid stereoisomers is different, probably because the net length of their isoprene tail varies depending on the presence and position of the kink introduced by the *cis* double bond. This observation is in agreement with recent publications that emphasize the role of membrane-mediated substrate and modulator interactions in the determination of the substrate spectrum of Pgp and ABCG2^41^.

To further analyze the interaction of retinoids with the transporters we studied their effects on the kinetic parameters of the substrate stimulated ATPase activity. For stimulation of ATPase activity we applied verapamil^42^,^43^ and quercetin^44^ that are real transported substrates of Pgp and ABCG2, respectively. Thus, in these experiments changes of the kinetic parameters in response to retinoids really reflect alterations in verapamil and quercetin binding and/or transport by the transporters. The apparent increase of the *K*_*M*_values of verapamil and quercetin (Figs 5 and 6 and Supplementary Fig. S4) in the presence of retinol and 13-*cis*-retinoic acid indicates direct interaction of retinol with both transporters and of 13-*cis-*retinoic acid with ABCG2. In addition, retinol and 13-*cis-*retinoic acid also decreased the *v*_*max*_ value of both transporters that might be either the result of non-competitive inhibition or of decreased effective concentration of the substrates (verapamil/quercetin) in the membrane. Non-competitive inhibition might be conveyed either by the binding of retinoids to an allosteric site of the transporter, or alternatively, brought about by the membrane rigidifying effect of retinoids. The latter explanation is in line with previous findings suggesting that lipid composition and membrane packing density in particular, can alter the function of Pgp probably by decreasing its conformational flexibility^45^,^46^. In addition, structural changes of the membrane may have an influence on the membrane partitioning and distribution of Pgp and ABCG2 substrates and thus their effective concentration in the vicinity of the drug binding site(s)^47^ (for a review see ref. 19). However, much further work will be required to fully understand these details.

In respect to the physiological relevance of our results, it seems unlikely that retinoids inhibit the examined ABC transporters expressed at various blood-tissue barriers, since their physiological tissue and blood concentration is in the nanomolar range (1–20 nM)^48^. However, retinoid therapy or retinol supplementation may result in sufficiently high local retinoid concentrations in the blood^24^,^25^,^26^,^49^, or in case of oral administration in the intestine that may block Pgp and ABCG2 expressed at organ-blood barriers or in the intestinal epithelium. Inhibition of the transporters may also affect the pharmacokinetics of other co-administered chemotherapeutic drugs. The above effects should be considered upon therapeutic application of retinoids to avoid drug-drug interactions occurring at the level of the membrane transporters, Pgp and ABCG2.

In conclusion, the different intramembrane orientation of certain retinoid stereoisomers might be important in their selective recognition by Pgp and ABCG2.

## Materials and Methods

### Chemicals

All chemicals, cell culture media and supplements were purchased from Sigma-Aldrich (Budapest, Hungary) except for all-*trans*-4-oxo-retinoic acid, which was kindly provided by DSM Nutritional products (Basel, Switzerland). Fluorescent dyes including calcein acetoxymethyl ester (calcein-AM) and Alexa 647 succinimidyl ester were purchased from Life Technologies, Inc. (Carlsbad, CA, USA). 5D3 and 15D3 mAbs were prepared from hybridoma supernatants using affinity chromatography and were >97% pure by SDS/PAGE. The 15D3 producing hybridoma cell line was obtained from the American Type Culture Collections (Manassas, VA, USA), while the 5D3 hybridoma was a kind gift from Brain P. Sorrentino (Division of Experimental Hematology, Department of Hematology/Oncology, St. Jude Children’s Research Hospital, Memphis, Tennessee, USA). The 5D3 and 15D3 antibodies were labeled with Alexa 647 succinimidyl ester (A647) and separated from the unconjugated dye by gel filtration on a Sephadex G-50 column. The dye-to-protein labeling ratio was around 3 for each antibody preparation. Stock solutions of lipophilic transporter substrates, modulators including calcein-AM, mitoxantrone, cyclosporin quercetin and retinoid derivatives were prepared in dimethyl-sulfoxide (DMSO). The final DMSO concentration in the samples was always less than 1% (v/v), and it did not have any effect on the transporters activity.

### Cell lines

The NIH 3T3 mouse fibroblast cell line and its human Pgp expressing counterpart (NIH 3T3 MDR1 G185^50^; kind gift from Michael Gottesman (National Institutes of Health, Bethesda, MD)) and the MDCK (Madin-Darby canine kidney) cell line and its ABCG2 transfected counterpart (kindly provided by Balazs Sarkadi (Institute of Enzymology, RCNS, Budapest)) were grown as monolayer cultures in Dulbecco’s modified Eagle’s medium (DMEM). Cell culture media were supplemented with 10% heat-inactivated fetal calf serum, 2 mM L-glutamine and 0.1 mg/ml penicillin-streptomycin cocktail. The cells were checked regularly for mycoplasma infection by the MycoAlert^®^ mycoplasma detection kit (Lonza Rockland Inc., Rockland, ME USA) and were found to be negative.

### Calcein and mitoxantrone accumulation tests

Calcein-AM was applied to measure the transport activity of Pgp^51^, while ABCG2 mediated transport was measured in mitoxantrone accumulation tests^18^. Cells were harvested and washed three times in PBS containing 7 mM glucose (gl-PBS). Cells (0.5 × 10^6^ ml^−1^ in gl-PBS) were pre-incubated in the presence or absence of the tested retinoids at different concentrations or specific inhibitors of Pgp (10 μM cyclosporin CsA) or ABCG2 (2 μM Ko143) for 20 min at 37 °C and then stained with 5 μM mitoxantrone for 40 min or 0.5 μM calcein-AM for another 20 min. The samples were washed three times with ice-cold PBS containing 0.5% FBS and kept on ice until flow cytometric measurement. Dead cells were excluded from the analysis on the basis of propidium iodide (PI) staining.

### Direct immunofluorescence

For detection of Pgp and ABCG2 cells (10^6^ ml^−1^ in gl-PBS) were incubated in the presence of 30 μg/ml Alexa647 conjugated 15D3 anti-Pgp mAb or 2 μg/ml 5D3 anti-ABCG2 mAb for 30 min at 37 °C. After two washes with ice-cold gl-PBS containing 1% bovine serum albumin (BSA-gl-PBS) expression levels of the transporters were measured by flow cytometry.

### Flow cytometry

Flow cytometric analysis was carried out on a Becton Dickinson FACSAria III Cell Sorter (Becton Dickinson, Mountain View, CA, USA). Calcein was excited by the 488 nm line of a solid state laser and the emitted light was detected using a 502 nm dichroic mirror and a 530/30 nm band-pass filter. PI was excited by the 562 nm line of a solid state laser and the emitted light was detected applying a 590 nm dichroic mirror and a 595/50 nm band-pass filter. AlexaFluor647 and mitoxantrone signals were detected with a 633 nm excitation laser and a 660/20 nm band-pass filter. Fluorescence signals were collected in logarithmic mode and the cytofluorimetric data were analyzed by the BDIS CELLQUEST (Becton Dickinson, Mountain View, CA, USA) software.

### Membrane preparations

For ATPase activity measurements we used membrane samples derived from Sf9 (*Spodoptera frugiperda*) insect ovarian cells expressing human Pgp or ABCG2. cDNAs of the transporters were cloned into recombinant baculovirus transfer vectors, the Sf9 insect ovarian cells were cultured, and infected with the baculoviruses as described in ref. 52. Sf9 cells overexpressing human Pgp and ABCG2 were harvested and cell membranes were isolated. The protein concentration of the membrane samples were determined by the Lowry method^53^. The ABCG2 expressing Sf9 membrane samples were loaded with cholesterol by cholesterol-methyl-beta-cyclodextrine complex in order to obtain high specific ATPase activity of the transporter^54^. Membrane samples were stored at −80 °C. The transporter expression of the membrane samples (5 μg membrane protein/slot) was routinely checked by immunoblot using the G-1 mAb (for Pgp) or BXP-21 mAb (for ABCG2) and a goat anti-mouse HRP-conjugated IgG secondary antibody. Both primary and secondary antibodies were from Santa Cruz Biotechnology Inc. (CA, USA) and applied at 1:5,000 dilution.

### ATPase activity measurements

The vanadate-sensitive ATPase activity of Pgp and ABCG2 was determined by a colorimetric assay described in refs 32,55. Specific ATPase activity of the transporters was calculated from the amount of released inorganic phosphate. The substrate dependent ATPase activity was stimulated by 40 μM verapamil in the Pgp expressing membrane samples, while the activity of ABCG2 transporters was induced by 10 μM quercetin. The absorbance of the samples was measured at 700 nm using a BioTek Synergy HT plate reader (BioTek Instruments, Winooski, USA).

The half-inhibitory doses (*IC*_*50*_) of retinoids were determined by fitting the dose-response relationships with a four parameter sigmoidal curve, where *A*_*max*_ and *A*_*min*_ are the ATPase activities measured at zero and at infinitely high inhibitor concentrations, respectively, *n* is the Hill coefficient and *x* is the concentration of the inhibitor:


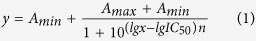


Upon studying the kinetics of inhibition of the substrate-stimulated ATPase activity by retinoids data points were fitted with a modified form of the Michaelis-Menten equation^56^ which includes the Hill coefficient, *n*:


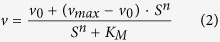


*v*_0_ is the basal ATPase activity measured in the absence of the stimulatory substrate, while *v*_max_ is the maximal extent of ATPase stimulation and *K*_*M*_ is the Michaelis constant (i.e. the substrate concentration at which the reaction rate is half of *v*_max_).

### Cellular uptake of retinoids and transporter inhibitors

NIH 3T3 mouse fibroblast cells (1 × 10^6^ cells/ml in gl-PBS containing 1% BSA were incubated with retinoids or transporter ligands (quercetin, cyclosporin Ko143) applied at different concentrations (1, 10 and 100 μM) for 30 min at 37 °C. After incubation the cells were removed by centrifugation at 400 × g. The cellular uptake of the examined compounds was determined by calculating the ratio of the absorbances measured in the supernatants at their absorption maximum before and after incubation with cells. Absorbances were determined using a NanoDrop 1000 UV/VIS Spectrophotometer (Thermo Fisher Scientific, Wilmington, DE, USA).

### Fluorescence anisotropy measurements

Cells (1 × 10^6^ cells/ml in Hank’s buffer (0.137 M NaCl, 5.4 mM KCl, 0.25 mM Na_2_HPO_4_, 0.44 mM KH_2_PO_4_, 1.3 mM CaCl_2_, 1.0 mM MgSO_4_, 4.2 mM NaHCO_3_, 7 mM glucose)) were pre-treated with 100 μM retinoid derivative for 10 min at 37 °C and then further incubated with 2 μM diphenylhexatriene (DPH) or 2 μM 1-[4-(trimethylammonio)phenyl]-6-phenyl-1,3,5-hexatriene (TMA-DPH) at room temperature, in dark for 20 min. Steady-state fluorescence anisotropy measurements were carried out at 37 °C using a Horiba Jobin Yvon Fluorolog-3 (Yvon Horiba, Edison, NJ, USA) spectrofluorimeter equipped with a thermostatted cell holder. The fluorescence of DPH and TMA-DPH was excited at 358 nm and their emission was measured at 427 nm. The steady-state fluorescence anisotropy of the dyes was calculated using the formula:


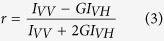


where, *I*_VV_ and *I*_VH_ are the vertically and horizontally polarized components of the fluorescence intensities, respectively, excited by vertically polarized light and *G* is a correction factor compensating for the unequal sensitivity of the detection system for vertically and horizontally polarized light. There is an inverse correlation between membrane fluidity and fluorescence anisotropy: the lower the anisotropy value, the higher the membrane fluidity; hence, the increase of fluorescence anisotropy is indicative of lower fluidity and higher structural order within the membrane^35^.

### Statistical analysis

Data were analyzed using SigmaStat (version 3.1, SPSS Inc., Chicago, IL, USA) and are presented as means ± SD. Comparison of two groups was carried out by unpaired *t*-test, statistical significance in the case of three or more groups was assessed using analysis of variance (ANOVA), applying the Holm-Sidak multiple comparison test for post hoc pair-wise comparison of the data. Differences were considered significant at P < 0.05. Dose-response curves were fitted using SigmaPlot 12.0 (SPSS Inc., Chicago, IL, USA).

## Additional Information

**How to cite this article:** Tarapcsák, S. *et al*. Interactions of retinoids with the ABC transporters P-glycoprotein and Breast Cancer Resistance Protein. *Sci. Rep.* **7**, 41376; doi: 10.1038/srep41376 (2017).

**Publisher's note:** Springer Nature remains neutral with regard to jurisdictional claims in published maps and institutional affiliations.

## Supplementary Material

Supplementary Information
