# Supplementary material for: Interactions of retinoids with the ABC transporters P-glycoprotein and Breast Cancer Resistance Protein
Source: Sci Rep. 2017 Feb 1;7:41376. doi: 10.1038/srep41376 (PMC5286421; doi:10.1038/srep41376)
Supplement: Supplementary Information [file srep41376-s1.pdf]

Title: Interactions of retinoids with the ABC transporters P-glycoprotein and Breast Cancer Resistance Protein

Running title: Interactions of retinoids with ABCB1 and ABCG2

Authors: Szabolcs Tarapcsák[1], Gábor Szalóki[1], Ágnes Telbisz[2], Zsuzsanna Gyöngy[1], Krisztina Matúz[3], Éva Csősz[3], Péter Nagy[1], Imre J. Holb[4,5], Ralph Rühl[6], László Nagy[3], Gábor Szabó[1] and Katalin Goda[1]

Author affiliations:

[1]Department of Biophysics and Cell Biology, University of Debrecen, Debrecen, H-4002 Egyetem tér 1, PO Box 400, HUNGARY

[2]Institute of Enzymology, Research Centre for Natural Sciences, Hungarian Academy of Sciences, Budapest, H-1117 Magyar tudósok körútja 2, PO Box 286, HUNGARY

[3]Department of Biochemistry and Molecular Biology, University of Debrecen, Debrecen, H-4002 Egyetem tér 1, PO Box 400, HUNGARY

[4]Institute of Horticulture, University of Debrecen, Debrecen, H-4015 Böszörményi út 138, P.O. Box 400, HUNGARY

[5]Plant Protection Institute, Centre for Agricultural Research, Hungarian Academy of Sciences, Budapest, H-1525 Hermann Ottó út 15, PO Box 525, HUNGARY

[6]MTA-DE, Public Health Research Group of the Hungarian Academy of Sciences, Faculty of Public Health, University of Debrecen, Debrecen, H-4028 Kassai út 26, PO Box 400, HUNGARY

Corresponding author: Katalin Goda, Department of Biophysics and Cell Biology, Faculty of Medicine, University of Debrecen. H-4002, Egyetem tér 1, Debrecen, HUNGARY; Tel.: (36) 52/412-623; Fax: (36) 52/532-201; P.O.B 400; E-mail: goda@med.unideb.hu

Keywords: retinoid derivatives, P-glycoprotein, ABCG2, ABC transporter, membrane fluidity, intramembrane localization

SUPPLEMENTARY FIGURES

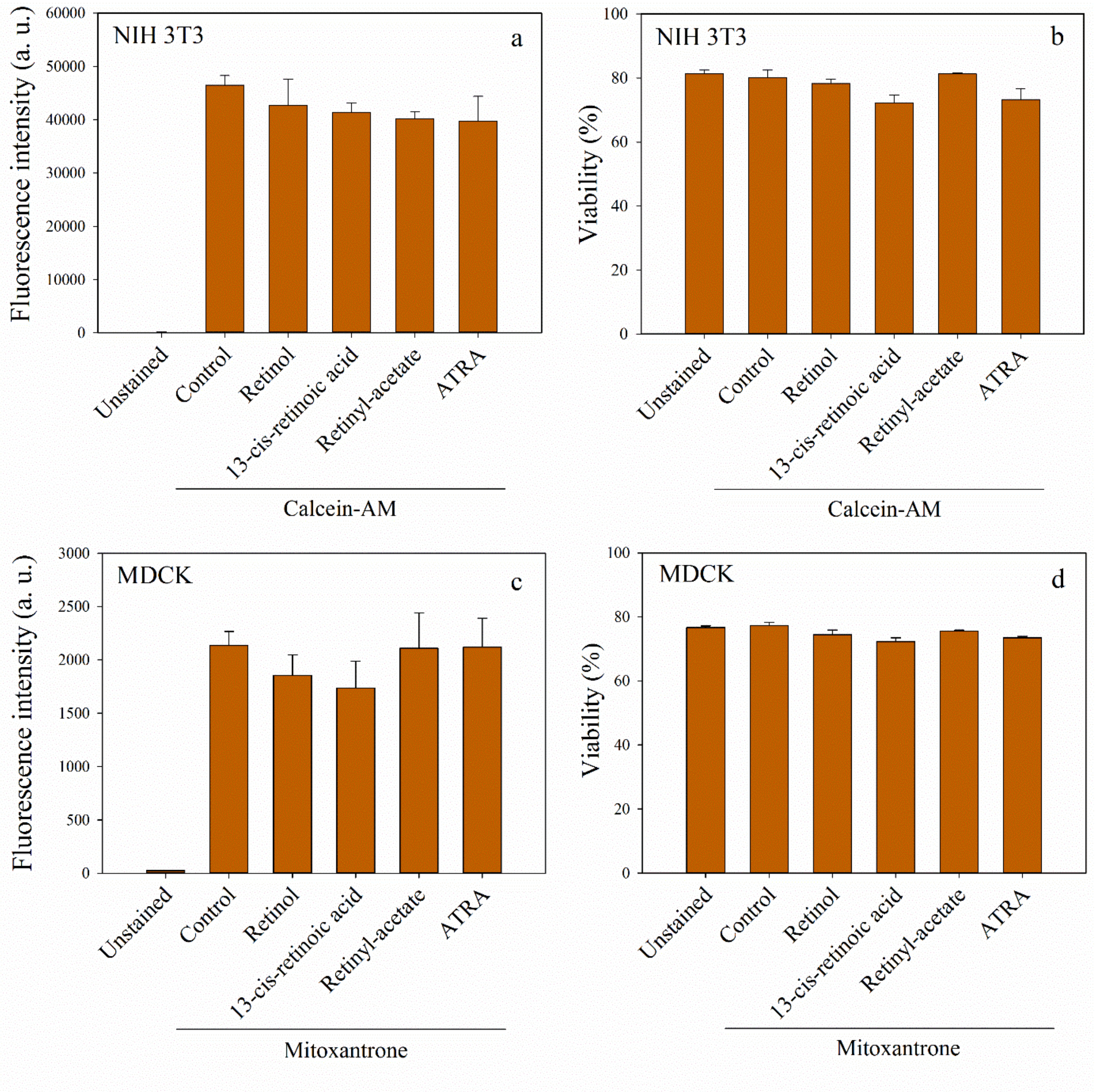

Supplementary Fig. S1: Effect of retinoid derivatives on the calcein (a) and mitoxantrone (c) accumulation and viability (b, d) of Pgp- and ABCG2-negative NIH 3T3 and MDCK cells. Cells were pre-treated with retinoids added at 50 µM concentration for 20 min at 37°C and then the NIH 3T3 cells were stained with 500 nM calcein-AM for 20 min (a), while the MDCK cells (b) were stained with 5 µM mitoxantrone for 40 min at 37°C. Cell viability was determined on the basis of propidium iodide exclusion.

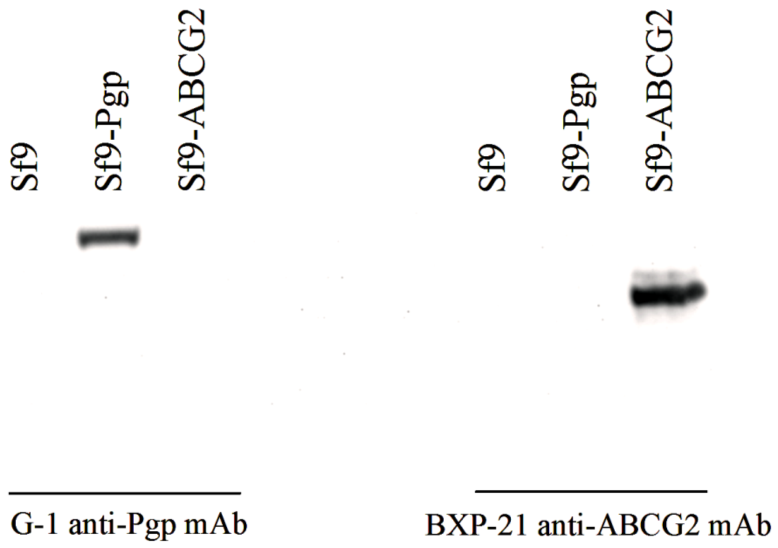

Supplementary Fig. S2: Western blot of membrane samples prepared from Sf9 cells expressing wild-type Pgp and ABCG2. Non-transfected Sf9 membrane sample is shown as a control. Preparation of membrane vesicles was carried out as described in Materials and Methods. The immunoblot was developed by the human Pgp-specific mouse monoclonal antibody G-1 and ABCG2-specific antibody BXP-21 (see Materials and Methods).

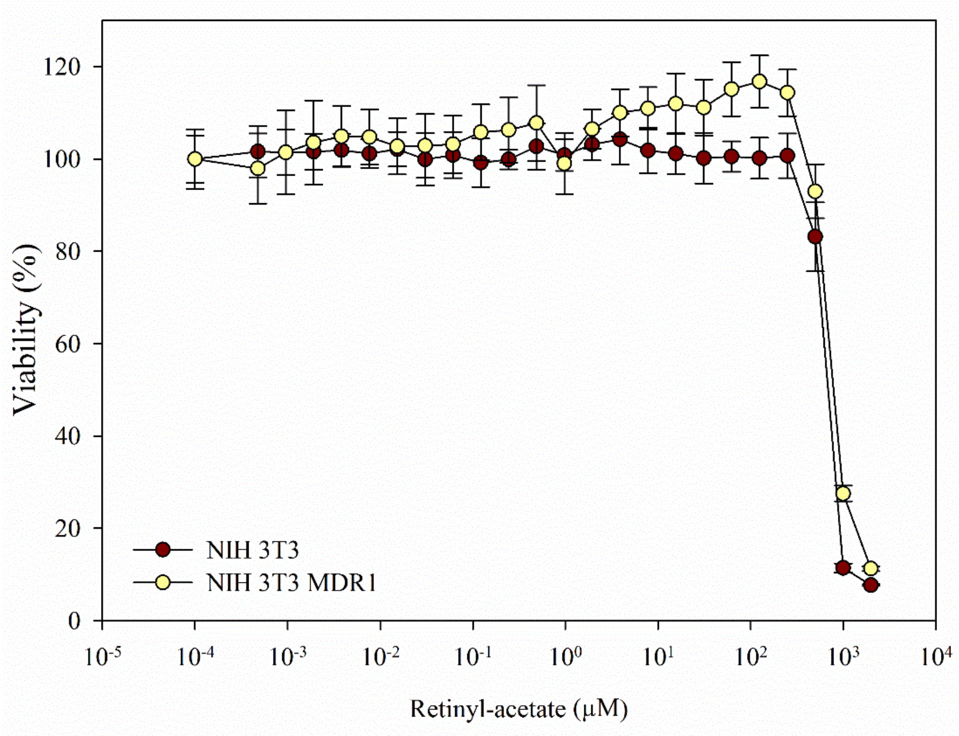

Supplementary Fig. S3: Cytotoxic effect of retinyl-acetate on Pgp-positive and Pgp-negative NIH 3T3 cells. The AlamarBlue (Serotec, UK) based *in vitro* cytotoxicity assay was performed as previously described [1]. Cells were seeded in 96-well plates at a cell density of 5 × 10^3 cells/well. 24 hours later retinyl-acetate was added at different concentrations and the plates were further incubated for 72 h at 37 °C. The cell viability was determined measuring the 530/590 nm fluorescence intensity of the dye in an automated microplate reader (BioTec Synergy HT, US). The AlamarBlue fluorescence intensity of the samples was normalized to the fluorescence of the retinyl-acetate untreated samples, and plotted as a function of retinyl-acetate concentration. The data points are means of eight parallel samples (±SD).

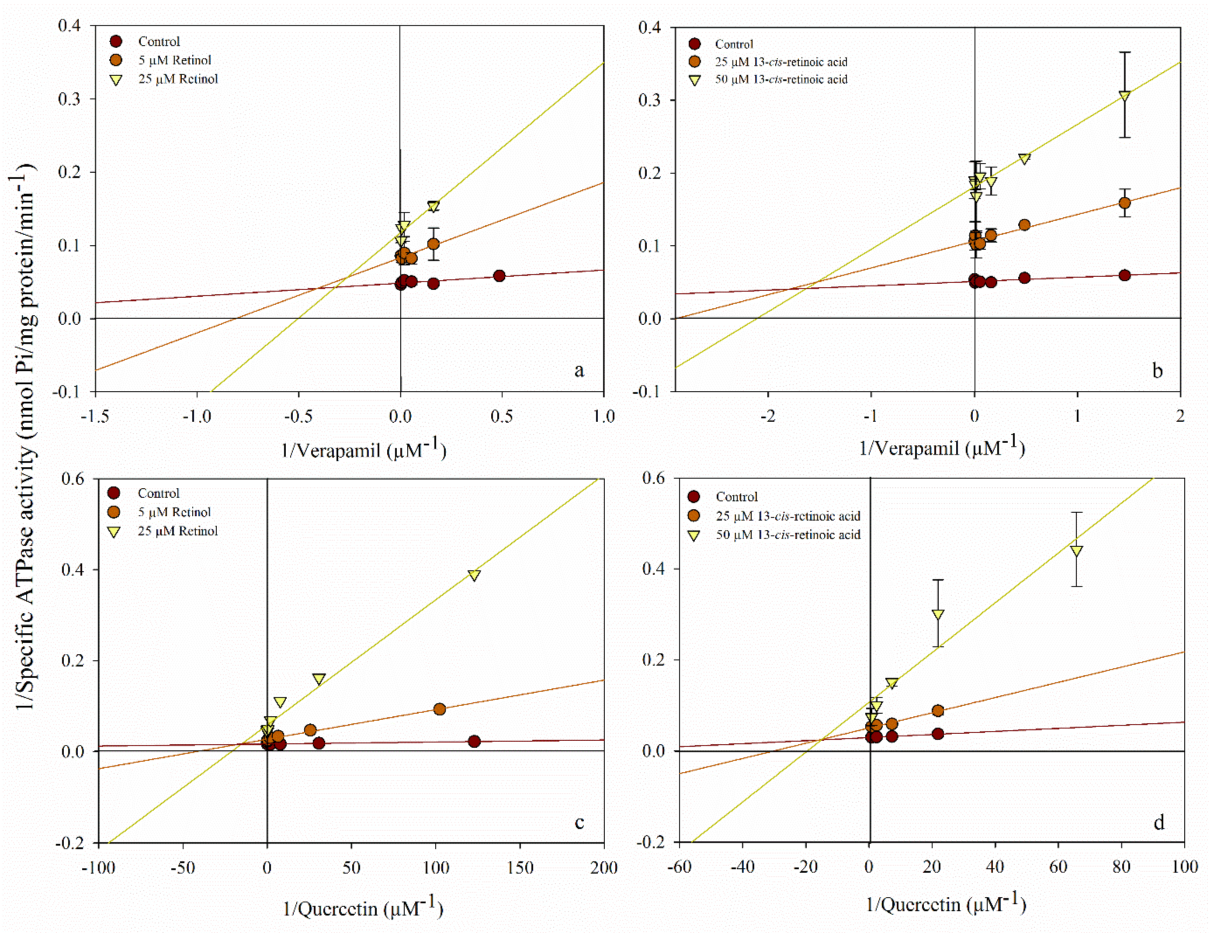

Supplementary Fig. S4: Lineweaver-Burk re-plots of the inhibition of the verapamil-stimulated ATPase activity of Pgp (a,b) and the quercetin-stimulated ATPase activity of ABCG2 (c,d) by retinol and 13-*cis*-retinoic acid. Verapamil and quercetin were applied at 50 µM and 10 µM concentrations, respectively. Representative data are shown out of three independent experiments (data points are means ± SD, n=3). The X-intercept of a Lineweaver-Burk plot is $-1/K_m$, while the Y intercept is $1/v_{max}$. Thus, retinoid treatments decrease v_{max} and increase K_m indicative of mixed-type inhibition (i.e. retinoids interfere with substrate binding *and* reduce the effectiveness of turnover).

SUPPLEMENTARY TABLES

IC$_{50}$ values (μM)				
Drug	Pgp		ABCG2	
	Basal activity	Stimulated activity	Basal activity	Stimulated activity
**Retinol**	23.5±4	27.9±0.7	31.6±4.2	95.2±10.6
**All-*trans*-retinoic-acid**	No effect	No effect	No effect	No effect
**9-*cis*-retinoic acid**	No effect	No effect	No effect	No effect
**13-*cis*-retinoic acid**	60.5±11.4	26.8±2	70.6±9	76.5±2.3
**All-*trans*-4-oxo-retinoic acid**	No effect	Weak inhibition at high conc.	No effect	No effect
**Retinyl-acetate**	36.8±4	19.1±5	18.9±4	70.4±7.3
**Retinyl-propionate**	No effect	No effect	No effect	No effect
**Retiny-palmitate**	No effect	No effect	No effect	No effect

Supplementary Table S1. Effects of retinoid derivatives on the ATPase activity of Pgp and ABCG2. The IC$_{50}$ values were calculated by fitting the dose-response curves with a four parameter logistic function ($R^2 \geq 0.98$, SigmaPlot 12.0).

Drug	LogP	Reference
**Quercetin**	1.8	[2]
**Cyclosporin A**	2.9	[3]
**Ko143**	3.5	CID 449171
**Retinol**	5.7	CID 445354
**All-*trans*-retinoic-acid**	6.3	CID 444795
**9-*cis*-retinoic acid**	6.3	CID 449171
**13-*cis*-retinoic acid**	6.3	CID 5282379
**All-*trans*-4-oxo-retinoic acid**	n. d.	n. d.
**Retinyl-acetate**	6.3	CID 638034
**Retinyl-propionate**	6.7	CID 6394572
**Retiny-palmitate**	13.6	CID 5280531

Supplementary Table S2. Octanol-water partition coefficients ($LogP_{ow}$ values) of the studied compounds. The values were collected from the National Center for Biotechnology Information. PubChem Compound Database Identification (CID) values are listed in the table.

PUBCHEM REFERENCES:

Ko143: National Center for Biotechnology Information. PubChem Compound Database; CID=10322450, https://pubchem.ncbi.nlm.nih.gov/compound/10322450 (Assessed Oct. 25, 2006)

Retinol: National Center for Biotechnology Information. PubChem Compound Database; CID=445354, https://pubchem.ncbi.nlm.nih.gov/compound/445354 (Assessed Sep. 16, 2004)

ATRA: National Center for Biotechnology Information. PubChem Compound Database; CID=444795, http://pubchem.ncbi.nlm.nih.gov/summary/summary.cgi?cid=444795 (Assessed Sep. 16, 2004)

9-*cis*-retinoic acid: National Center for Biotechnology Information. PubChem Compound Database; CID=449171, http://pubchem.ncbi.nlm.nih.gov/summary/summary.cgi?cid=449171 (Assessed Mar. 25, 2005)

13-*cis*-retinoic acid: National Center for Biotechnology Information. PubChem Compound Database; CID=5282379, http://pubchem.ncbi.nlm.nih.gov/summary/summary.cgi?cid=5282379 (Assessed Jun. 24, 2005)

Retinyl-acetate: National Center for Biotechnology Information. PubChem Compound Database; CID=638034, http://pubchem.ncbi.nlm.nih.gov/summary/summary.cgi?cid=638034 (Assessed Mar. 27, 2005)

Retinyl-palmitate: National Center for Biotechnology Information. PubChem Compound Database; CID=5280531, http://pubchem.ncbi.nlm.nih.gov/summary/summary.cgi?cid=5280531 (Assessed Sep. 16, 2004)

Retinyl-propionate: National Center for Biotechnology Information. PubChem Compound Database; CID=6394572, http://pubchem.ncbi.nlm.nih.gov/summary/summary.cgi?cid=6394572 (Assessed Oct. 07, 2005)

REFERENCES FOR SUPPLEMENTARY INFORMATION

1 Szaloki, G. *et al.* The strong in vivo anti-tumor effect of the UIC2 monoclonal antibody is the combined result of Pgp inhibition and antibody dependent cell-mediated cytotoxicity. *PloS one* **9**, e107875, doi:10.1371/journal.pone.0107875 (2014).

2 Rothwell, J. A., Day, A. J. & Morgan, M. R. Experimental determination of octanol-water partition coefficients of quercetin and related flavonoids. *Journal of agricultural and food chemistry* **53**, 4355-4360, doi:10.1021/jf0483669 (2005).

3 el Tayar, N. *et al.* Solvent-dependent conformation and hydrogen-bonding capacity of cyclosporin A: evidence from partition coefficients and molecular dynamics simulations. *Journal of medicinal chemistry* **36**, 3757-3764 (1993).
